# Piezoresistive Membrane Surface Stress Sensors for Characterization of Breath Samples of Head and Neck Cancer Patients

**DOI:** 10.3390/s16071149

**Published:** 2016-07-22

**Authors:** Hans Peter Lang, Frédéric Loizeau, Agnès Hiou-Feige, Jean-Paul Rivals, Pedro Romero, Terunobu Akiyama, Christoph Gerber, Ernst Meyer

**Affiliations:** 1 Department of Physics, University of Basel, Swiss Nanoscience Institute, Klingelbergstrasse 82, CH-4056 Basel, Switzerland; Christoph.Gerber@unibas.ch (C.G.); Ernst.Meyer@unibas.ch (E.M.); 2 SAMLAB, EPFL Neuchâtel, CH-2002 Neuchâtel, Switzerland; frederic.loizeau@sensirion.com (F.L.); akiyama@nanoworld.com (T.A.); 3 Department of Otolaryngology—Head and Neck Surgery, CHUV, University of Lausanne, CH-1015 Lausanne, Switzerland; Agnes.Hiou@chuv.ch (A.H.-F.); Jean-Paul.Rivals@chuv.ch (J.-P.R.); 4 Ludwig Cancer Research Center, Department of Fundamental Oncology, Faculty of Biology and Medicine, University of Lausanne, CH-1066 Epalinges, Switzerland; Pedro.Romero@hospvd.ch

**Keywords:** piezoresistive membrane sensors, surface stress sensor, nanomechanical sensor, electronic nose, breath analysis, head and neck cancer

## Abstract

For many diseases, where a particular organ is affected, chemical by-products can be found in the patient’s exhaled breath. Breath analysis is often done using gas chromatography and mass spectrometry, but interpretation of results is difficult and time-consuming. We performed characterization of patients’ exhaled breath samples by an electronic nose technique based on an array of nanomechanical membrane sensors. Each membrane is coated with a different thin polymer layer. By pumping the exhaled breath into a measurement chamber, volatile organic compounds present in patients’ breath diffuse into the polymer layers and deform the membranes by changes in surface stress. The bending of the membranes is measured piezoresistively and the signals are converted into voltages. The sensor deflection pattern allows one to characterize the condition of the patient. In a clinical pilot study, we investigated breath samples from head and neck cancer patients and healthy control persons. Evaluation using principal component analysis (PCA) allowed a clear distinction between the two groups. As head and neck cancer can be completely removed by surgery, the breath of cured patients was investigated after surgery again and the results were similar to those of the healthy control group, indicating that surgery was successful.

## 1. Introduction

More than a century ago, medical practitioners asked patients to exhale in order to figure out whether their breath contained specific smells possibly related to a particular disease. This old idea is here adopted to investigate breath samples of cancer patients using a nanomechanical electronic nose device. Specific chemical tracer substances or chemical by-products of metabolic processes are often found in the patient’s breath for many diseases of the respiratory tract system. Conventionally, breath samples are analyzed using gas chromatography and mass spectrometry methods, but interpretation of results is difficult and time-consuming. Here, an electronic nose technique is presented to characterize patients’ exhaled breath samples in a non-invasive way which allows a simpler analysis than with the abovementioned classical standard analytical procedures.

Cancer is a disease where cells are growing in an uncontrolled way forming a tumor, invading and destroying adjacent healthy tissues and organs. Cancerous cells can spread to other locations in the body via lymph or blood vessels to form metastases, the most common cause of cancer-related death in patients with solid tumors. 

Head and neck squamous cell carcinoma (HNSCC) is the fifth most important cancer type worldwide. HNSCC is highly curable if detected early. However, second primary tumors and local recurrences are a major challenge, the latter being the most common cause of treatment failure and disease-related death. Early detection of HNSCC and identification of residual or recurrent disease in treated patients allow early therapeutic intervention and may result in a survival advantage. Diagnosis is normally performed by endoscopy and taking a biopsy of suspect lesions. We propose here a non-invasive diagnostic technique based on detection of volatile organic compounds (VOCs) in exhaled breath using an electronic nose technique.

Detection of head and neck cancer using patients’ exhaled breath is a well-established technique [1,2]. In cancer progression, the squamous cells of the head or neck provoke cellular oxidative stress [3], leading to the emission of cancer-specific VOCs into the blood [4]. A part of the VOC biomarkers in the blood are transmitted to the alveolar exhaled breath through exchange via the lung. The presence of such VOCs (particularly straight and monomethylated alkanes and benzene derivatives) in breath is documented by gas chromatography/mass spectrometry measurements [5,6], These types of VOCs also occur in the breath of healthy subjects, but in a different composition ratio as in cancer patients [4]. Numerous reports on successful application of electronic noses for breath testing have been reported in the literature for many years [7,8,9,10,11,12,13,14,15].

In recent years, mechanics has experienced a revival, as microfabrication technologies and nanotechnology are applied to produce tiny structures. The development started with a novel imaging technique named atomic force microscopy [16], which provides ultrahigh resolution of a surface on the atomic scale. This technology is based on raster-scanning a surface with a microfabricated cantilever beam that has a tiny tip at its free end. While keeping the distance constant between tip and surface by controlling their interaction force, a topography map of the surface is produced, revealing details on the atomic scale. Most frequently, a laser is used to determine the tiny deflection of the cantilever in the nanometer range by reflecting the laser beam at the apex of the cantilever and measuring the position with a lateral photodiode. Although the cantilever is very small, the readout still requires tabletop-sized equipment.

A cantilever beam is an excellent force sensor for ultra-small forces in the nano-Newton range. The high sensitivity can not only be used for atomic force microscopy, but also allows one to apply the cantilever beam for measuring surface forces during molecular adsorption processes on the cantilever surface, thus enabling cantilevers as chemical sensors [17].

Over the years, cantilever sensors have turned out to be very useful for detecting DNA hybridization with single point mutation sensitivity [18], protein and antibody recognition [19], and even for assessing patient eligibility for cancer treatment [20]. The only drawback is that the equipment for optical cantilever deflection readout is still quite bulky. This disadvantage can be overcome by employing a different method for deflection detection, namely the use of piezoresistor elements to determine bending. The required readout electronics then fits in a portable box of 10 cm × 10 cm × 16 cm, including data acquisition and gas handling. For detection of head and neck cancer, we take here advantage of the bending responses of an array of piezoresistive polymer-coated membranes due to exposure to VOCs. 

## 2. Materials and Methods 

### 2.1. Microfabrication

**Figure 1:**
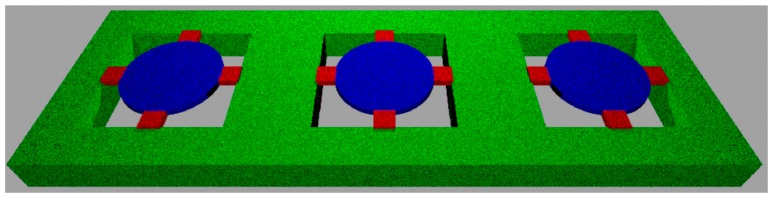
Schematic representation of an array of membrane-type surface stress sensor (MSS). The actual diameter of the round membrane (shown in blue) is 500 µm and its thickness is 2.5 µm. The membrane is suspended by four sensing beams with integrated p-type piezoresistors (shown in red), representing a full Wheatstone bridge. A solid supporting frame (green) holds the sensor.

Medical applications favor the routine use of a compact, small-sized, portable and non-invasive device. A prototype was used to examine patients’ exhaled breath samples in search for VOC patterns associated with head and neck cancer. Membrane-type surface stress sensors (MSS) have been first described by Yoshikawa et al. [21]. Their application for medical sensing have been reported in by Loizeau et al. [22,23]. MSS arranged in arrays for molecular detection in gaseous phase have been microfabricated from silicon-on-insulator substrates and structured by deep reactive ion etching. The round membranes have a diameter of 500 µm and a thickness of 2.5 µm and are suspended by four sensing beams with integrated p-type piezoresistors, representing a full Wheatstone bridge (Figure 1). The p-doped piezoresistors have been fabricated using two distinct doping processes (ion diffusion through boron silica glass and implantation). The latter method features shallow resistors, which are very sensitive to surface stress changes.

### 2.2. Membrane Functionalization

**Figure 2:**
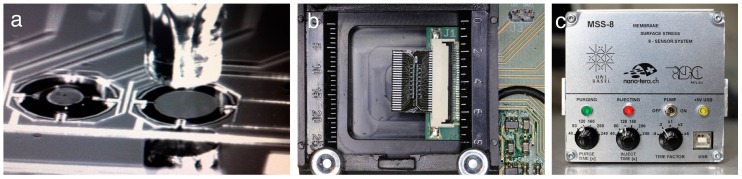
(**a**) Each membrane is coated with a different polymer that responds by swelling in a characteristic way to surrounding molecules. Functionalization of MSS is done using inkjet spotting of polymer solutions in water (10 mg/mL); (**b**) MSS are arranged in arrays for detection of VOCs in a gas stream passing through the measurement chamber. The numbers on the left indicate the scale in millimeters. (**c**) Portable universal serial bus powered compact measurement device with pumping system for gaseous samples, signal readout and data acquisition.

The membranes have been coated with a thin (<1 µm) polymer layer using inkjet spotting (Figure 2). VOCs present in the breath sample will diffuse into the polymer layer in a way characteristic for each polymer resulting in swelling [14] and produce bending of the membrane. As eight different polymers are used, a characteristic bending pattern of the membranes is generated on exposure to an individual patient’s breath samples. The polymers applied are carboxymethyl cellulose (CMC), poly-(2-ethyl-2-oxazoline) (PEO), polyethylene glycol methyl ether methacrylate macromer (PEGMEMA), hydroxypropyl cellulose (HPC) poly(acrylic acid)-acetic acid (PAA-AA), poly-(vinylpyridine) (PVPy), butyl rubber (PIB), and polyethylenimine (PEI).

### 2.3. Clinical Pilot Study

In a clinical pilot study, we investigated breath samples from head and neck cancer patients and healthy donors (smokers) as control persons in a double blind trial. The patient inclusion criteria were based on histologically confirmed carcinoma at a comparable stage. Exclusion criteria of patients to the study population were the following:

1. Previous history of squamous cell carcinoma of the lung or upper aerodigestive tract.
2. Synchronous lesions of another histological type.
3. Heart disease (NYHA Class III or IV).
4. Serious pathology, such as infections requiring antibiotic use, uncontrolled peptic ulcers, gastrointestinal bleeding, central nervous system disorders.
5. History of immunodeficiency or autoimmune pathologies.
6. Metastases in the central nervous system, not treated and progressing.
7. Chemotherapy, radiotherapy, immunotherapy less than 4 weeks prior to entry into the study (6 weeks for nitrosoureas).
8. Concomitant treatment with steroids and antihistamines. Topical or inhaled steroid application is allowed.
9. Psychiatric disorders or dependencies that could prevent informed consent.
10. Kidney dysfunction with creatinine >2× upper limit of normal value.
11. Diabetes.

The patients were selected from the same age groups (between 60 and 85 years old, male and female). The clinical pilot study comprised unfortunately only a few usable patient samples. Originally there were four healthy donors, and nine head and neck squamous cell carcinoma patients. From the nine head and neck squamous cell carcinoma patients, there were only three who provided useable breath samples before and after surgery. For the other six there was either only a breath sample before surgery available or the volume of the sample was too small to allow reliable measurements (in some cases only 0.2 L was provided, which does not allow one to reliably follow the measurement protocol). Patients and donors were asked to breathe into a 1 liter Tedlar bag. Breath samples were collected before surgery and after surgery, typically 2 weeks after the operation. The bags were then stored at 4 °C until analysis. Each breath sample has been measured seven times, whereby the first injection-purge cycle has been discarded to avoid influence of previous measurements.

## 3. Results and Discussion

**Figure 3:**
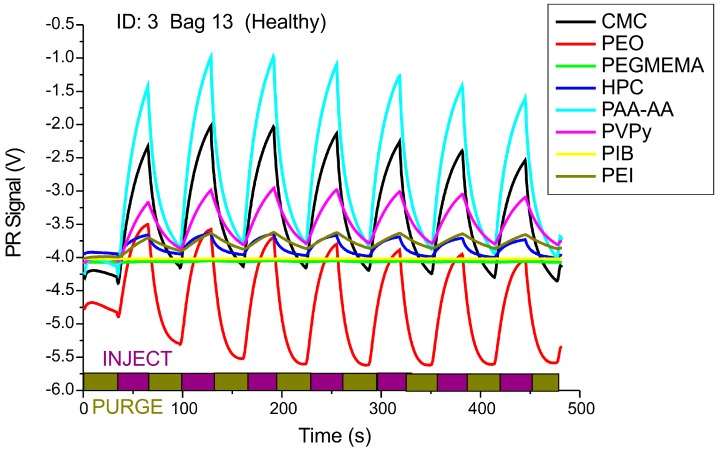
Piezoresistive (PR) membrane response curves upon injection with patients’ breath samples and purging with dry nitrogen. Injection and purging duration: 30 s, flow rate 15 mL/min.

Breath samples from head and neck cancer patients and healthy donors (smokers) have been characterized by the MSS electronic nose technique. Gaseous sample from the Tedlar sample bag were transported into the measurement chamber by pumping at a rate of 15 mL/min using micropumps (Bartels Mikrotechnik GmbH, Dortmund, Germany). After exposure to the polymer-coated MSS sensors for 30 s, the chamber has been purged with dry nitrogen gas also for 30 s. Several injection/purge cycles have been performed, resulting in a chart as shown in Figure 3. Polymer-coated membranes can be reproducibly regenerated by purging them with dry nitrogen gas.

**Figure 4:**
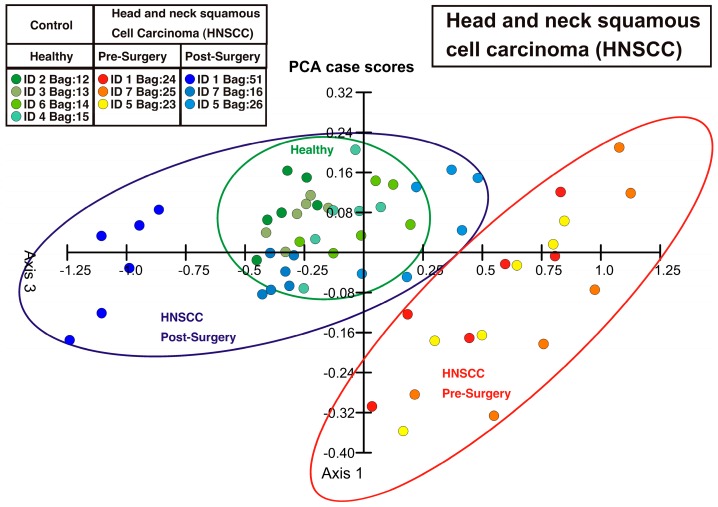
Principal component analysis (PCA) plot showing three distinct clusters (indicated with ellipses) that represent healthy control persons, HNSCC patients before surgery and HNSCC patients after surgery, i.e., after removal of the tumor by operation. The points of the HNSCC patients after surgery are at a similar location in the PCA plot as those from the healthy persons and differ clearly from the points of the HNSCC patients before surgery, indicating that the removal of the tumor has been successful.

The deflection values within an injection/purge cycle at 10, 15, 20 and 25 s after beginning of each injection were subtracted from the value at the beginning of the injection (0 s) to reduce the influence of possible drifts in the measurement. These four differential values obtained for each membrane sensor were processed using principal component analysis (PCA) to be represented in a two-dimensional plot showing one dot for each breath sample measurement, i.e., one injection-purging cycle (Figure 4).

**Figure 5:**
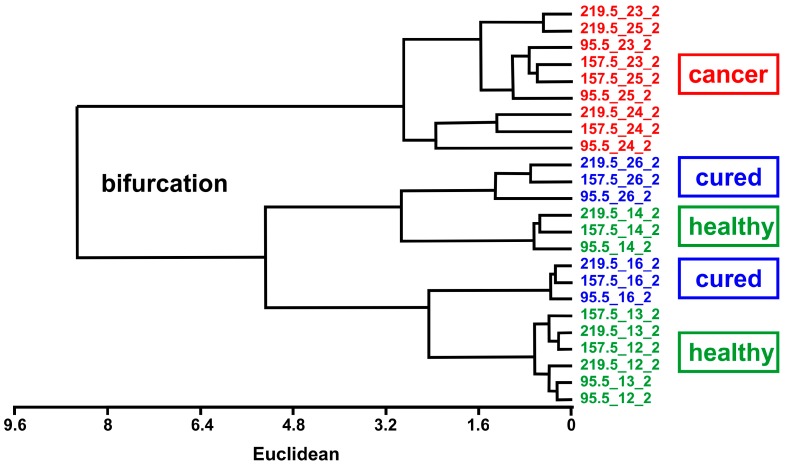
The UPGMA diagram (dendrogram) shows bifurcations for distinct distances between pairs of measurements implying that the datasets from cancer patients (HNSCC) before surgery are clearly different from healthy control persons and cured NHSCC patients after surgery. Number labels indicate individual injection-purge cycles.

To emphasize the distinction capability of the method, hierarchical tree analysis (unweighted pair group method with arithmetic mean, UPGMA) was performed. In this method, the data are analyzed by calculating the Euclidian distance between vectors consisting of data points and their closest neighbors. Figure 5 shows the UPGMA diagram of the data. Breath measurements of NHSCC patients before surgery are clearly different from measurements of healthy control persons and cured NHSCC patients after surgery, demonstrating the success of surgery.

Other evidence that VOC profiles in exhaled breath can be used to detect diseases has been shown by Phillips et al. for lung and breast cancer [24]. A pilot study of analysis of air exhaled by HNSCC patients using an array of five gold nanoparticle sensors and gas chromatography has shown promising results [1,2]. VOCs related to diseases like diabetes mellitus and uraemia in breath were reported to be detectable easily using polymer-coated nanomechanical cantilever arrays [25], allowing to distinguish different VOCs.

Screening for HNSCC using tests or diagnostic methods using a non-invasive method based on exhaled breath represents a major advantage for possible detection of tumors at an early stage. Identification of subjects that showed indication of VOCs related to HNSCC allows subsequent closer examination using invasive traditional techniques like extraction of a tissue sample (biopsy). The detection of tumors based on testing the exhaled breath of patients, is especially promising for tumors of the upper aerodigestive tract, as the probability is high that VOCs originating from different metabolic pathways of the cell, in particular from tumors at an early stage, can be detected.

## 4. Conclusions

Early detection of primary tumors and of recurrences after surgical removal of the primary tumor is crucial for patients with HNSCC. Invasive analyses, e.g. endoscopies, give clear indication on the treatment success, but are a hassle for the patient. Detecting the VOCs in exhaled breath represents a non-invasive method to follow the success of a treatment/surgery. We have shown that MSS are capable to distinguish HNSCC patients before surgery from healthy control persons and HNSCC patients after surgery by monitoring VOCs in patients’ breath samples. The measurement device used is portable and powered by a laptop computer’s universal serial bus port.

Detecting VOCs associated with cancer growth will ultimately lead to a simple, easily performable and non-invasive screening technique that can be used in conjunction with, or as alternative to standard more invasive techniques. The technique could eventually be adapted to other pathologies affecting the respiratory tract.

## Abbreviations

The following abbreviations are used in this manuscript:

MSS	Membrane Surface stress Sensor
HNSCC	Head and Neck Squamous Cell Carcinoma
PCA	Principal Component Analysis
PR	Piezoresistive
UPGMA	Unweighted Pair Group Method with Arithmethic Mean
VOC	Volatile Organic Compound

## References

[1] Hakim M., Billan S., Tisch U., Peng G., Dvrokind I., Marom O., Abdah-Bortnyak R., Kuten A., Haic H. (2011). Diagnosis of head-and-neck cancer from exhaled breath. Brit. J. Cancer.

[2] Gruber M., Tisch U., Jeries R., Amal H., Hakim M., Ronen O., Marshak T., Zimmerman D., Israel A.E., Doweck I. (2014). Analysis of exhaled breath for diagnosing head and neck squamous cell carcinoma: A feasibility study. Brit. J. Cancer.

[3] Okunieff P., Fenton B., Chen Y. (2005). Past, present, and future of oxygen in cancer research. Adv. Exp. Med. Biol..

[4] Schmutzhard J., Rieder J., Deibl M., Schwentner I.M., Schmid S., Lirk P., Abraham I., Gunkel A.R. (2008). Pilot study: Volatile organic compounds as a diagnostic marker for head and neck tumors. Head Neck.

[5] Gordon S.M., Szidon J.P., Krotoszynski B.K., Gibbons R.D., O’Neill H.J. (1985). Volatile organic-compounds in exhaled air from patients with lung-cancer. Clin. Chem..

[6] Phillips M., Cataneo R.N., Cummin A.R.C., Gagliardi A.J., Gleeson K., Greenberg J., Maxfield R.A., Rom W.N. (2003). Detection of lung cancer with volatile markers in the breath. Chest.

[7] Konvalina G., Haick H. (2014). Sensors for breath testing: From nanomaterials to comprehensive disease detection. Acc. Chem. Res..

[8] Broza Y.Y., Haick H. (2013). Nanomaterial-based sensors for detection of disease by volatile organic compounds. Nanomedicine.

[9] Haick H., Broza Y.Y., Mochalski P., Ruzsanyi V., Amann A. (2014). Assessment, origin, and implementation of breath volatile cancer markers. Chem. Soc. Rev..

[10] Vishinkin R., Haick H. (2015). Nanoscale sensor technologies for disease detection via volatolomics. Small.

[11] Peled N., Barash O., Tisch U., Ionescu R., Broza Y.Y., Illouze M., Mattei J., Bunn P.A., Hirsch F.R., Haick H. (2013). Volatile fingerprints of cancer specific genetic mutations. Nanomed. Nanotechnol. Biol. Med..

[12] Barash O., Peled N., Tisch U., Bunn Jr. P.A., Hirsch F.R., Haick H. (2012). Classification of lung cancer histology by gild nanoparticle sensors. Nanomed. Nanotechnol. Biol. Med..

[13] Broza Y.Y., Kremer R., Tisch U., Gevorkyan A., Shiban A., Best L.A., Haick H. (2013). A nanomaterial-based breath test for the short-term follow-up after lung tumor resection. Nanomed. Nanotechnol. Biol. Med..

[14] Bachar N., Liberman L., Muallem F., Feng X., Müllen K., Haick H. (2013). Sensor arrays based on polycyclic aromatic hydrocarbons: Chemoresistors versus quartz-crystal microbalance. ACS Appl. Mater. Interfaces.

[15] Nakhleh M.K., Amal H., Awad H., Gharra A., Abu-Sahel N., Jeries R., Haick H., Abassi Z. (2014). Sensor arrays based on nanoparticles for early detection of kidney injury by breath samples. Nanomed. Nanotechnol. Biol. Med..

[16] Binnig G., Quate C.F., Gerber C. (1986). Atomic force microscope. Phys. Rev. Lett..

[17] Baller M.K., Lang H.P., Fritz J., Gerber C., Gimzewski J.K., Drechsler U., Rothuizen H., Despont M., Vettiger P., Battiston F.M. (2000). A cantilever array-based artficial nose. Ultramicroscopy.

[18] Fritz J., Baller M.K., Lang H.P., Rothuizen H., Vettiger P., Meyer E., Güntherodt H.-J., Gerber C., Gimzewski J.K. (2000). Translating biomolecular recognition into nanomechanics. Science.

[19] Backmann N., Zahnd C., Huber F., Bietsch A., Plückthun A., Lang H.P., Güntherodt H.-J., Hegner M., Gerber C. (2005). A label-free immunosensor array using single-chain antibody fragments. Proc. Natl. Soc. Sci. USA.

[20] Huber F., Lang H.P., Backmann N., Rimoldi D., Gerber C. (2013). Direct detection of a BRAF mutation in total RNA from melanoma cells using cantilever arrays. Nat. Nanotechnol..

[21] Yoshikawa G., Akiyama T., Gautsch S., Vettiger P., Rohrer H. (2011). Nanomechanical membrane-type Surface Stress Sensor. Nano Lett..

[22] Loizeau F., Akiyama T., Gautsch S., Vettiger P., Yoshikawa G., de Rooij N. Membrane-type surface stress sensor with piezoresistive readout. Proceedings of the 26th European Conference on Solid-State Transducers.

[23] Loizeau F., Lang H.P., Akiyama T., Gautsch S., Vettiger P., Tonin A., Yoshikawa G., Gerber Ch., de Rooij N. Piezoresistive membrane-type surface stress sensors arranged in arrays for cancer diagnosis through breath analysis. Proceedings of the 26th IEEE International Conference on Micro Electro Mechanical Systems (MEMS 2013).

[24] Phillips M., Altorki N., Austin J.H., Cameron R.B., Cataneo R.N., Greenberg J., Kloss R., Maxfield R.A., Munawar M.I., Pass H.I. (2007). Prediction of lung cancer using volatile biomarkers in breath. Cancer Biomark..

[25] Schmid D., Lang H.P., Marsch S., Gerber Ch., Hunziker P. (2008). Diagnosing disease by nanomechanical olfactory sensors-system design and clinical validation. Eur. J. Nanomed..

